# Pregnancy and pregnancy intention after experiencing infertility: A longitudinal study of women in Malawi

**DOI:** 10.1371/journal.pgph.0001646

**Published:** 2023-11-14

**Authors:** Marta Bornstein, Alison Gemmill, Alison H. Norris, Sarah Huber-Krum, Jessica D. Gipson

**Affiliations:** 1 Division of Epidemiology, Ohio State University College of Public Health, Columbus, Ohio, United States of America; 2 Division of Health Promotion, Education, and Behavior, University of South Carolina Arnold School of Public Health, Columbia, South Carolina, United States of America; 3 Johns Hopkins Bloomberg School of Public Health, Department of Population, Family and Reproductive Health, Baltimore, Maryland, United States of America; 4 Ohio State University College of Social Work, Columbus, Ohio, United States of America; 5 UCLA Fielding School of Public Health, Department of Community Health Sciences, Los Angeles, Los Angeles, California, United States of America; University of California San Francisco (UCSF), UNITED STATES

## Abstract

**Background:**

Infertility is a common experience among individuals and couples. Infertility may resolve without intervention, but little is known about pregnancy intentions and incidence of pregnancy following infertility, particularly in low-resource settings.

**Methods:**

Data come from UTHA, a longitudinal cohort study in Central Malawi, with baseline and follow up surveys conducted from 2014–2019 (N = 1,030 reproductive-aged women). We assessed bivariable and multivariable relationships between reported infertility at baseline and subsequent pregnancy and retrospective pregnancy intentions. Pregnancy intention was measured with the London Measure of Unplanned Pregnancy (LMUP), a scale validated in Malawi (Range = 0–12).

**Results:**

Approximately 20% of the sample reported that they had ever experienced infertility (tried to become pregnant for at least two years without conceiving in that time) at baseline. The proportion of women who reported a new pregnancy during the follow up period (mean = 4.3 years) was the same (65%) for women who had and had not experienced infertility. Among women who became pregnant, levels of pregnancy intendedness were similar between women who had and had not experienced infertility. Prospective desire for a/another child at baseline was associated with subsequent pregnancy (AOR: 1.59; 95%CI: 1.06–2.39) and was also associated with higher levels of pregnancy intendedness measured retrospectively (LMUP of 9.4 vs. 8.4).

**Conclusions:**

Experienced infertility was not associated with differential odds of having a subsequent pregnancy or the intendedness of a subsequent pregnancy. Thus, women who have experienced infertility should be included in family planning programs and research to support all women in achieving their reproductive goals.

## Introduction

Both infertility (i.e., the inability to become pregnant after 1–2 years of trying) and unintended fertility are common, yet there is limited research that acknowledges the possibility that people may experience both infertility and unintended fertility within their reproductive life course [1]. Despite concurrently high rates of infertility and total fertility in population-level estimates, the relationship between experiences with infertility and subsequent intended and unintended fertility have seldom been investigated longitudinally within individuals. Understanding fertility experiences after infertility provides a holistic and dynamic view of the reproductive life course, improving upon existing work that focuses on singular reproductive events or experiences.

Coexisting high rates of infertility and unintended pregnancy are evident in Malawi, where approximately 20% of women report infertility (“*have you ever tried to conceive a pregnancy for two years or longer without conceiving in that time*?*”*) [2] or difficulty becoming pregnant (“*whether an individual reports ever experiencing a difficult time in getting pregnant”*) [3] and 41% of pregnancies are considered unintended [4]. Much remains unknown about the fertility experiences of women who become pregnant after experiencing a period of infertility or subfecundity, especially in lower-resource settings. Moreover, studies suggest that women who have experienced infertility may be less likely to use contraception than women who have not experienced infertility [5–7]. If women who have experienced infertility decide they do not want to become pregnant, there may be an opportunity to intentionally include them in family planning programs to support all women in reaching their reproductive goals.

Studies that examine the incidence of pregnancy after an experience of infertility are often focused on the clinical phenomenon of becoming pregnant after a one- or two-year period of exposure to unprotected sex. While the majority (79–92%) of fecund women will become pregnant within one year of attempting to become pregnant [8, 9], studies in clinical settings have found that around half of women who met the clinical definition for infertility–no pregnancy after one year–eventually became pregnant without infertility treatment [8, 10–12]. These studies demonstrate the potential to experience a period of infertility and still be able to conceive a pregnancy at some point within the reproductive life course. Women who become pregnant after meeting the definition for experiencing infertility are sometimes referred to as having resolved infertility or are referred to as “truly fertile.” Unlike sterility, which is not addressed in our paper, infertility refers to a specific period, not necessarily a permanent state; thus, it is possible that unintended fertility could follow a period of infertility. In this study, we refer to all women who have ever met the definition of infertility reflected in the survey item (having tried to conceive for two years without conceiving in that time) as having ever experienced infertility.

No studies, to our knowledge, explicitly examine pregnancy desires among women who become pregnant after experiencing infertility. There is a common assumption that women who experience infertility want to become pregnant, even though this may not be an accurate or enduring assumption [13]. Fertility preferences are dynamic [14], perhaps even among women and couples who once tried to become pregnant for a 1–2-year period and were not able to. Understanding the potential for women to have an *unintended* pregnancy after experiencing infertility presents challenges because it requires retrospective or longitudinal data that ideally spans the reproductive life course to understand the temporality of infertility and unintended fertility, as well as possible linkages between the two experiences.

Studying infertility and unintended fertility requires nuanced and time-specific measures of both phenomena. Moreover, both infertility and unintended fertility are difficult to measure [14–16]. Thus, few studies have examined the occurrence of unintended pregnancy after experiencing infertility, although one study found that having a “later-than-expected” birth—potentially due to fertility problems—may be associated with increased odds of a subsequent mistimed birth, likely due to the assumption that it would take longer to conceive based on their previous experience [17].

**Fig 1:**
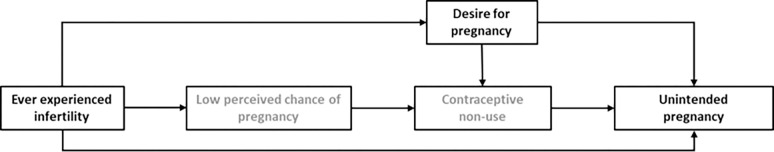
Analytic framework examining ever experienced infertility, pregnancy, and unintended pregnancy^1^. 1Constructs in grey are not examined in this study.

A deeper understanding of fertility after experiencing infertility is critical to ensuring that family planning programs address the needs of women and couples, both while they are experiencing difficulty becoming pregnant, as well as after (if they eventually conceive). A proposed mechanism for the relationship between experienced infertility and unintended pregnancy is shown in Fig 1, informed by constructs within the Health Belief Model [18]. Ever experiencing infertility may lower one’s perceived risk of becoming pregnant, contributing to contraceptive non-use, which in turn may lead to unintended pregnancy. Of course, women who experience infertility may want to become pregnant (and, indeed, may only report infertility if they want to become pregnant), which would potentially influence both contraceptive use and pregnancy [13]. Other women may be infertile, but because they are not actively trying to become pregnant, they may be unaware or unlikely to be counted as infertile in some measures [13]. Additionally, women who have experienced infertility may eventually report that they do not desire to become pregnant as a way of resolving the cognitive dissonance between what one wants and what one believes they can realistically achieve [19]. Indeed, infertility has been associated with lower fertility intentions in a U.S. study [20].

This study aims to answer two main questions: (1) independent of pregnancy desire, are women who ever experienced infertility, here defined as ever trying to conceive for two years or longer without conceiving in that time, less likely to become pregnant in the future than women who have not experienced infertility? And, (2) among women who become pregnant, are those who have experienced infertility more likely to report that their pregnancy was unintended compared to women who had not experienced infertility?

## Methods

### Data

Data for this study come from the *Umoyo Wa Thanzi* (UTHA; Health for Life) research program, a cohort study focused on sexual and reproductive health decision making among women and their male partners in Central Malawi. The cohort was recruited from the catchment area of a rural, non-profit hospital after a complete household census of the area. The 68 villages within the catchment area were collapsed into 43 clusters based on size, and 11 clusters (19 villages) were randomly selected for inclusion in the cohort [21].

All women of reproductive age residing in the 11 clusters were eligible to participate. The overall response rate was 96% in Wave 1 (2014–2015). Subsequent data collection occurred in Wave 2 (2015), Wave 3 (2016–2017), Wave 4 (2017–2018), and Wave 5 (2019). Using Wave 1 as the reference point, there was an 83% retention rate between Waves 1 and 3; 76% between Waves 1 and 4; and 63% between Waves 1 and 5. It is not appropriate to calculate a retention rate between Waves 1 and 2, as Wave 2 included only a subset of participants from the cohort and is excluded from this study. Data were collected using tablets in the participant’s home or another private space by Malawian research assistants. Participants were compensated with 1,500 MK in Waves 1–3 and 2,000 MK in Waves 4 and 5 (approximately 1.50–2.00 U.S. dollars).

### Ethics

Participants gave verbal and written consent and re-consented at each wave of data collection. Participants under the age of 18 years were eligible if they were married and therefore legally able to consent for themselves. At the time of data collection, laws in Malawi permitted marriage under age 18 years. This study was approved by the Institutional Review Boards (IRB) at The Ohio State University (IRB number 2013B0172), University of California–Los Angeles (IRB number 18–000879), the Malawi College of Medicine.

### Analytic samples

#### Pregnancy incidence sample–participated in Wave 1 and at least one additional wave

The first analysis, which assesses the incidence of pregnancy, includes women who participated in Wave 1 of the UTHA cohort (2014–2015) and at least one additional wave of data collection (2016–2017, 2017–2018, and/or 2019). Of the 1,030 women who participated in Wave 1, women who were sterilized (n = 24), over the age of 40 years (n = 4), or never had sex (n = 77) were excluded, resulting in a total of 925 eligible women. An additional 94 women were excluded because they did not participate in any follow up waves. Finally, 55 women were excluded because they had missing data for a key Wave 1 covariate (e.g., number of pregnancies as of Wave 1, ever experienced infertility at Wave 1), resulting in a total sample of 776 women.

#### Pregnancy sample

To examine pregnancy intention/planed-ness of a pregnancy following Wave 1, our denominator only included women who became pregnant between Wave 1 (2014–2015) and Wave 5 (2019) (N = 503). Because retrospective pregnancy intentions/planning were only assessed at Wave 5, 128 of these women who did not participate in Wave 5 were excluded. Thus, the pregnancy sample includes 375 women.

#### Sample selectivity

Multiple imputation to address missing data was considered, but ultimately not pursued because women frequently had missing data on multiple variables, including the variable used to construct the main outcome (number of pregnancies at Wave 1). Comparing the pregnancy incidence sample (N = 776) to those who were excluded because they did not participate in a follow up wave (n = 94) or because they had missing data (n = 55), we noted several important differences. A larger proportion of women in the analytic sample were married or cohabiting at Wave 1 than those who were excluded due to non-follow up or missing data (92% vs. 70%). The analytic sample was also older on average (26.6 vs. 25.5 years). Women who were excluded from the analytic sample were more likely to be nulliparous than women in the analytic sample (24% vs. 7%) and had fewer living children (30% of women in the excluded sample had no living children compared to 10% in the analytic sample) (S1 Table). These differences are likely due to the increased mobility of women who are younger, not married, or do not have children, making them more likely to have relocated outside of the UTHA cohort area during the study period. Similar patterns emerged when comparing the pregnancy analytic sample (N = 375) to those who were excluded because they did not participate in Wave 5 (n = 128) (S2 Table).

### Variables

**Dependent variables. Any new pregnancy after Wave 1** was calculated by subtracting the number of lifetime pregnancies women reported at Wave 1 from the number they reported at each subsequent wave. This variable was then recoded as binary (1 indicated that they *ever* reported a new pregnancy after Wave 1 and 0 indicated that they reported no new pregnancies after Wave 1). The outcome of new pregnancy was examined in both samples.

Number of pregnancies was chosen rather than number of living children because living children may fluctuate due to child mortality. Thus, reporting fewer living children after Wave 1 is possible and cannot be assumed to be a reporting error [22]. Complete birth histories were not available.

**Retrospective pregnancy planning** was measured using the London Measure of Unplanned Pregnancy (LMUP) among women who had at least one pregnancy between Waves 1–5 (the pregnancy sample). The authors of the LMUP use the terms ‘unintended’ and ‘unplanned’ interchangeably [23]. This six-item scale was developed by a research team who conducted qualitative research and psychometrically tested and validated the scale in a sample of women in western Europe (Cronbach’s alpha = 0.97; test/retest reliability = 0.97) [23]. It was later validated in Malawi (Cronbach’s alpha = 0.78; rest/retest reliability = 0.80) [24]. The scale includes items measuring “expressed intentions, desire for motherhood, contraceptive use, pre-conceptual preparations, personal circumstances/timing, and partner influences” [23]. An English translation of the Chichewa version of the LMUP used in Malawi, along with how each response option is scored, is included in S3 Table. As suggested by the authors of the LMUP scale, we constructed the LMUP as a continuous variable, with scores ranging from 0 (least planned/intended) to 12 (most planned/intended) [23].

In the present study, all six LMUP items were correlated and the entire scale yielded a Cronbach’s Alpha of 0.90 (not shown). Item six (pre-conception preparations) was the least correlated with other items, but all six items loaded onto a single factor, and thus all six items were retained.

#### Independent variables

All independent variables were measured at Wave 1 (2014–2015). The main independent variable was **ever experienced infertility** *(have you ever tried to conceive a pregnancy for two years or longer without conceiving in that time*?*)*. We examined additional sociodemographic covariates typically associated with incidence of pregnancy and/or pregnancy intentions, including relationship status, age, number of pregnancies and number of living children, and education [21, 22, 25] (Table 1).

**Table 1:** Independent variables, response options (measured at Wave 1).

**Construct/survey item**	**Response options and coding**
**Ever experienced infertility**^**1**^ (*Have you ever tried to conceive a pregnancy for two years or longer without conceiving in that time*?*)*	Don’t know (0) No (0) Yes (1)
**Relationship status**	Not married or cohabiting (0) Married/cohabiting (1)
**Age group**	15–19 years 20–24 years 25–29 years 30–34 years 35–40 years
**Sexually Transmitted Infection (STI) history** *(In your life*, *have you ever had an STI (for example*: *syphilis*, *herpes*, *gonorrhea*, *and chlamydia)*?*)*	Don’t know (0) No (0) Yes (1)
**Number of living children**	**Continuous:** 0–8 children
**Desire for a/another child** *(Would you EVER like to have a/another child*?*)*	No (0) Yes (1) Undecided (1)^2^
**Years of education**	**Continuous:** 0–12 years
**Years in cohort** ^**3**^	**Continuous:** 1.7–5.1 years

^1^Ever experienced infertility is examined as both an independent and dependent variable in bivariable analyses. In multivariable models, ever experienced infertility is used as an independent variable only.

^2^11 participants were undecided about having more children at the time of the survey and are coded as ‘yes.’ Dropping these participants or coding the response as ‘no’ did not impact the results.

^3^The length of time in the cohort varied based on how many and which follow up waves a participant was surveyed. A longer time under observation would increase the odds of reporting a pregnancy, and thus length of time in the cohort was included as an independent variable. To construct the length of time in the cohort, the date of the participant’s first survey (sometime between 2014–2015) was subtracted from the date of the participant’s last survey (sometime between 2016–2019).

### Analysis

The independent variables were measured at Wave 1 and the dependent variables (reporting a pregnancy after Wave 1 and intention of most recent pregnancy) were measured using Waves 3–5 to establish temporality in the main relationships of interest. In the pregnancy incidence sample, we examined bivariable associations between sociodemographic characteristics and the focal independent variable (ever experienced infertility) and bivariable associations between sociodemographic characteristics and the dependent variables of interest: reporting a pregnancy after Wave 1. In the sample of women who became pregnant, we examined bivariable associations between sociodemographic characteristics and intention of most recent pregnancy among those who reported a new pregnancy after Wave 1.

We used chi^2^ tests of independence and F-tests to assess differences in distributions and means. We then constructed a multivariable logistic regression model examining the odds of reporting a new pregnancy after Wave 1, controlling for pregnancy desires and sociodemographic characteristics, in the pregnancy incidence sample. We also adjusted for number of years in the cohort to account for different lengths of follow-up. In an alternative approach, we conducted survival analyses to assess potential differences in time-to-conception between women who did and did not report ever experiencing infertility at Wave 1 among a sub-sample of women who participated in Waves 1, 3, 4, and 5 (N = 477).

In our sample used to assess pregnancy intentions/planning, we examined pregnancy intention/planning of the most recent pregnancy using the LMUP. The purpose of examining the LMUP only among women who became pregnant between Waves 1 and 5 was to ensure that only pregnancies that occurred after reporting ever experiencing infertility were included. We used one-way ANOVAs to examine crude association between sociodemographic characteristics, including infertility, and the LMUP as a continuous outcome. Two participants did not respond to one of the six LMUP items; thus, their scores were imputed based on their responses to the other five items.

### Study setting and cohort makeup

The study took place in a rural area of Central Region Malawi. The predominant ethnic group in this area is Chewa and nearly all speak Chichewa. Most residents in the area were subsistence farmers who earned less than the equivalent of 2 USD per day. Very few had access to piped water (4%) and 30% lived in a home with a metal roof [2, 26]. Malawi Demographic and Health Survey (DHS) data reports that around 14% of households have access to piped water to their home or yard and that about 50% of Malawian households have a metal roof [4].

There are other notable differences between the UTHA cohort and Malawi as a whole. Compared to DHS data, our cohort was slightly younger (mean age = 26.5 in UTHA vs. 28.1 in DHS) and included a narrower range of ages (15–40 years in UTHA vs. 15–49 years in DHS). Our sample also was less likely to be nulliparous (10% of the UTHA sample vs. about a quarter of women in the DHS). A greater proportion of women in the UTHA cohort wanted more children (71%) compared to in the DHS (about 56%) [4]. Just over half (56%) of women in the cohort were using a form of modern contraception at the first wave of data collection (2014). The DHS similarly reported that 58% of married women were using a form of modern contraception (2015–2016) [4].

## Results

### Pregnancy incidence sample

Table 2 shows characteristics of women who had and had not ever experienced infertility reported at Wave 1. Overall, 65% of women became pregnant after Wave 1; the proportion was the same for women who had experienced infertility (65%) and for women who had not experienced infertility (65%) (Table 2). Women who reported infertility at Wave 1 were more likely to have ever experienced a sexually transmitted infection (STI) than women who did not report infertility (16% vs. 9%; p<0.05) and had fewer years of education on average (4.3 vs. 5.4 years; p<0.001). Notably, there were no meaningful differences by age, number of pregnancies, or number of living children at Wave 1 between women who did and did not report infertility (Table 2).

**Table 2:** Wave 1 characteristics of women by ever experiencing infertility at Wave 1 (pregnancy incidence sample; N = 776)1.

	Total	No infertility at W1 N = 623	Infertility at W1 N = 153	p-value
	%/mean (range)	%/mean (range)	%/mean (range)	
**New pregnancy reported after W1**				NS
Yes	64.8%	64.9%	64.7%	
No	35.2%	35.2%	35.3%	
**Relationship status**				NS
Married/cohabiting	92.1%	91.7%	94.1%	
Not married or cohabiting	7.9%	8.4%	5.9%	
**Age (mean (range))**	26.6 (15–40)	26.4 (15–39)	27.2 (15–40)	NS
**Age group**				NS
15–19	12.3%	12.0%	13.1%	
20–24	31.6%	33.1%	25.5%	
25–29	21.4%	21.0%	22.9%	
30–34	20.0%	21.4%	18.3%	
35–40	14.8%	13.5%	20.3%	
**STI history**				<0.05
Yes	10.4%	9.2%	15.7%	
No	89.6%	90.9%	84.3%	
**Number of pregnancies (mean (range))** ^**2**^	3.1 (0–12)	3.1 (0–12)	3.1 (0–7)	
**Number of pregnancies**				NS
None	7.2%	6.9%	8.5%	NS
1	16.6%	16.9%	15.7%	
2	20.0%	20.4%	18.3%	
3	17.8%	18.0%	17.0%	
4+	38.4%	37.9%	40.5%	
**Number of living children (mean (range))**	2.5 (0–8)	2.5 (0–8)	2.3 (0–6)	NS
**Number of living children**				NS
None	10.3%	9.3%	14.4%	
1	20.2%	20.4%	19.6%	
2	24.6%	24.6%	24.8%	
3	18.3%	18.5%	17.7%	
4+	26.6%	27.3%	23.5%	
**Desire for a/another child (ever)**				NS
Yes	70.8%	70.1%	73.2%	
No	29.2%	29.9%	26.8%	
**Years of education (mean (range))**	5.2 (0–12)	5.4 (0–12)	4.3 (0–12)	<0.001
**Years in cohort (mean (range))**	4.3 (1.7–5.1)	4.3 (1.7–5.1)	4.3 (2.0–5.1)	NS

^1^p-values are for Chi^2^ tests (categorical variables) and F tests (continuous variables); NS = not significant.

^2^Used to compute outcome

Table 3 shows characteristics of women who did and did not report a new pregnancy after Wave 1. Approximately 20% of both women who did and did not report a pregnancy after Wave 1 had ever experienced infertility as reported in Wave 1. Women who became pregnant after Wave 1 were younger than women who did not (26 vs. 28 years as of Wave 1; p<0.001), had experienced fewer pregnancies by Wave 1 (2.6 vs. 3.9; p<0.001), and had fewer living children (2.2 vs. 3.0; p<0.001) at Wave 1. Additionally, women who reported that they wanted a/nother child at Wave 1 were more likely to report becoming pregnant after Wave 1 (77% vs. 59%; p<0.001) (Table 3).

**Table 3:** Wave 1 characteristics of women who reported a new pregnancy after Wave 1 (pregnancy incidence sample; N = 776)1.

	No pregnancy (N = 273)	Pregnancy (N = 503)	p-value
	%/mean (range)	%/mean (range)	
**Infertility**			NS
Yes	19.8%	19.7%	
No	80.2%	80.3%	
**Relationship status**			NS
Married/cohabiting	92.7%	91.9%	
Not married or cohabiting	7.3%	8.1%	
**Age (mean (range))**	28.2 (15–40)	25.7 (14–39)	<0.001
**Age group**			<0.001
15–19	7.7%	14.7%	
20–24	22.0%	36.8%	
25–29	26.0%	18.9%	
30–34	24.9%	17.3%	
35–40	19.4%	12.3%	
**STI history**			NS
Yes	13.2%	9.0%	
No	86.8%	91.0%	
**Number of pregnancies (mean (range))** ^**2**^	3.9 (0–12)	2.6 (0–10)	<0.001
**Number of pregnancies**			<0.001
None	0.7%	10.7%	
1	8.8%	20.9%	
2	16.5%	21.9%	
3	20.2%	16.5%	
4+	53.9%	30.0%	
**Number of living children (mean (range))**	3.0 (0–7)	2.1 (0–8)	<0.001
**Number of living children**			<0.001
None	5.1%	13.1%	
1	13.2%	24.1%	
2	22.3%	25.8%	
3	23.1%	15.7%	
4+	36.3%	21.3%	
**Desire for a/another child (ever)**			<0.001
Yes	59.3%	76.9%	
No	40.7%	23.1%	
**Years of education (mean (range))**	5.1 (0–12)	5.2 (0–12)	NS
**Years in cohort (mean (range))**	4.1 (1.7–5.1)	4.3 (2.1–5.1)	<0.01

^1^p-values are for Chi^2^ tests (categorical variables) and F tests (continuous variables); NS = not significant.

^2^Used to compute outcome

Next, we present results from logistic regressions estimating odds of reporting a new pregnancy after Wave 1. In the unadjusted model, ever experienced infertility was not associated with reporting a pregnancy after Wave 1 (OR = 0.99; 95% CI: 0.69–1.44), and this relationship was similar in the adjusted model (AOR = 0.89; 95% CI: 0.60–1.34) (Table 4). The 95% confidence intervals for older age groups, number of living children, desire for a/another child, and years in the cohort did not include 1.0 in the unadjusted models (Table 4).

**Table 4:** Odds of reporting a new pregnancy after Wave 1 (N = 776).

	Bivariable		Multivariable	
	OR	95% CI	AOR	95% CI
**Ever experienced infertility**	0.99	0.69–1.44	0.89	0.60–1.34
**Married/cohabiting**	0.89	0.51–1.55	0.91	0.50–1.68
**Age group**				
15–19	ref		ref	
20–24	0.88	0.50–1.54	1.02	0.56–1.85
25–29	0.38	0.21–0.67	0.62	0.31–1.21
30–34	0.36	0.20–0.65	0.87	0.41–1.83
35–40	0.33	0.18–0.61	0.95	0.41–2.20
**History of an STI**	0.65	0.41–1.03	0.75	0.45–1.23
**# Of Living children**	0.74	0.68–0.81	0.77	0.65–0.90
**Desire for a/another child (ever)**	2.29	1.66–3.14	1.59	1.06–2.39
**Years of education**	1.00	0.96–1.05	0.95	0.90–1.01
**Years in cohort**	1.39	1.17–1.64	1.51	1.26–1.81

In the adjusted model, number of living children at Wave 1 was associated with lower odds of reporting a pregnancy (AOR = 0.77; 95% CI: 0.65–0.90). Desire for a/another child at Wave 1 (AOR: 1.59; 95% CI: 1.06–2.39) and years in the cohort (AOR: 1.51; 95% CI: 1.26–1.81) were both associated with higher odds of reporting a pregnancy (Table 4).

In survival analyses examining the relationship between infertility history and time-to-conception during the follow-up period, we find no noteworthy differences between women who had experienced infertility and those who had not, supporting our results from the logistic regression approach (S4 Table and S1 Fig).

### Pregnancy intentions/planning

Among the 375 women who became pregnant between Waves 1 and 5, we examined the six items that comprise the LMUP scale individually (S5 Table) and as a composite continuous measure, with higher scores indicating the pregnancy was more intended. Overall, pregnancies in the cohort tended to be intended. The average LMUP score among the sample was 9.13 (median: 10; range: 0–12), and this did not differ between women who experienced infertility and those who did not. When we examined individual items, we found that the majority (91%) of women were not using contraception when they became pregnant (item 1). Approximately 80% of women reported that their pregnancy happened at the right time (item 2), that they intended to get pregnant (item 3), that they wanted to have a baby (item 4), and that they agreed with their partner to have a baby before becoming pregnant (item 5). Finally, 35% of women engaged in a pro-active behavior prior to becoming pregnant (e.g., eating healthily, seeking advice) (item 6). There were no significant differences in any of the individual LMUP items by whether or not a woman reported ever experiencing infertility at Wave 1.

Examining the relationships between participant characteristics and mean LMUP score, we found that women who wanted a/another child at Wave 1 tended to have higher LMUP scores than women who did not report wanting a/another child at Wave 1 (9.4 vs. 8.4; p<0.05) (Table 5). We also found that women who had experienced infertility tended to have slightly lower LMUP scores (i.e., had slightly lower pregnancy intendedness) than women who had not experienced infertility (8.8 vs. 9.2), but this difference was not statistically significant (Table 5). Women in older age groups tended to score slightly lower on the LMUP than younger woman (e.g., the mean LMUP for women aged 25–29 years was 9.7 and the mean for women aged 35–40 years was 8.5), but this difference was also not statistically significant. Given lack of statistical significance and relatively large standard deviations, indicating a lack of meaningful variation in LMUP scores by experienced infertility and most other sociodemographic characteristics, we did not conduct a multivariable linear regression predicting the LMUP score.

**Table 5:** Mean LMUP score among women who became pregnant by Wave 5 (N = 375).

	N	Mean LMUP score	Std. Dev.	p-value
**Average LMUP score**	375	9.13 (0–12)	3.50	
**Infertility**				NS
Yes	69	8.81	3.62	
No	306	9.21	3.48	
**Relationship status**				NS
Married/cohabiting	348	9.12	3.54	
Not married or cohabiting	27	9.26	3.06	
**Age group**				NS
15–19	46	9.48	3.22	
20–24	140	9.28	3.41	
25–29	74	9.70	2.93	
30–34	64	8.45	4.04	
35–40	51	8.45	3.91	
**STI history**				NS
Yes	28	10.04	2.40	
No	347	9.06	3.57	
**Number of pregnancies**				NS
None	30	9.00	3.47	
1	77	9.78	2.87	
2	83	9.20	3.44	
3	61	9.43	3.49	
4+	124	8.57	3.86	
**Number of living children**				NS
None	39	9.30	3.12	
1	87	9.48	3.19	
2	103	9.40	3.31	
3	62	9.16	3.61	
4+	84	8.33	4.04	
**Desire for a/another child (ever)**				<0.05
Yes	285	9.35	3.33	
No	90	8.43	3.95	
**Years of education**				NS
0–5 years	236	8.98	3.49	
6–12 years	139	9.40	3.52	

## Discussion

This study found that experiencing infertility, measured here by not becoming pregnant after two years of trying, may not indicate sustained infertility or permanent sterility. Two-thirds of women in this study population became pregnant during the follow up period, regardless of whether they reported at Wave 1 ever having experienced infertility. We also found no meaningful differences in the retrospective consideration of pregnancy intendedness between women who did and did not report ever having experienced infertility at Wave 1.

There are many possible explanations for the lack of difference in subsequent incidence of pregnancy between women who ever experienced infertility and those who had not. First, we consider how those who reported infertility at Wave 1 differed from those who did not report infertility. Notably, the 20% of women who reported infertility at Wave 1 had largely demonstrated their ability to become pregnant, indicated by the fact that nearly all (92%) of the women who reported ever experiencing infertility reported that they had been pregnant at least once. Almost as many (86%) had at least one living child. Neither number of pregnancies nor number of living children at Wave 1 differed meaningfully between those who had ever experienced infertility and those who had not. A limitation of this study is that the measure of infertility does not specify *when* a woman experienced a period of two years trying to become pregnant without conceiving in that time. It is possible that women experienced infertility prior to their first pregnancy and had since been able to conceive one or more pregnancies, and it is also possible that women experienced infertility when trying to conceive a second or higher order pregnancy, or that they were currently experiencing infertility at the time of the Wave 1 survey. Because this timing was not specified, we could not control for possible differences in reports of a pregnancy by timing or duration of experienced infertility.

Some of our findings may also be driven by the make-up of our sample, as only two women in the analytic sample reported zero pregnancies throughout the observation period. Women who were excluded from the analytic sample because they lacked follow up data were younger, less likely to be married, and more likely to be nulliparous at Wave 1 than women in the analytic sample. It is possible that these women were less likely to become pregnant than those with follow up data, but are not captured because they relocated outside of the UTHA cohort area for marriage, perhaps because of divorce or remarriage, which we know may be a consequences of experiencing infertility [27]. In comparing our sample to the DHS, we found that our sample of women was both younger on average and less likely to be nulliparous compared to the DHS sample of women. The DHS does not include a direct question about infertility. Thus, we cannot make any direct comparisons regarding how infertility may differ in our sample.

Although the two-year definition of infertility used in this study surpasses the threshold required for a clinical diagnosis of infertility and the WHO definition of infertility (one year without conceiving) [28], the data indicate that some women may have experienced infertility episodically. A possible explanation for episodic infertility may be the presence of active sexually transmitted infections (STIs) that are treated before causing clinical Pelvic Inflammatory Disease (PID), or permanent scarring to a woman’s reproductive system [29]. While STI progression may lead to PID and infertility, women with STIs that are treated before clinical PID develops may experience reduced fecundity, or a period where they try unsuccessfully to become pregnant, but are ultimately able to conceive once treated for the STI [30]. Trends in our data support this: at Wave 1, women who reported that they had experienced infertility were more likely to report that they had ever experienced an STI than women who did not report infertility. Integrating a the full spectrum of sexual and reproductive health services, such as HIV/STI prevention, screening, and treatment, along with family planning, infertility, and maternal health, may have a variety of positive impacts on reproductive health and well-being, including infertility prevention [31].

The rate of pregnancy following a report of ever experiencing infertility in our study is similar to what has been found in a study in New Zealand where, among cases of untreated infertility or subfertility in women, approximately half spontaneously conceived within three years [11]. Given the lack of available infertility treatment in Malawi and in similar low resource settings [32–34], it is likely that women who reported infertility and then conceived a pregnancy did so without biomedical treatment for infertility, although we cannot confirm this because use of treatment was not measured.

Infertility is typically experienced at the couple-level, but the measure used in this study does not distinguish between whether a woman was reporting a history of male or female infertility (and, in many cases, she may not know). There are challenges to being able to understand the biological source or cause of infertility within a couple outside of a clinical study setting, but we know that women may be held responsible for infertility due to social and gender norms [35]. A recent review (2020) found that 21% of infertility is attributable to male factors alone and an additional 20% is attributable to both female and male factors in the Africa region [36]. In our study population, the origin of infertility may not be known, but previous research suggests that women and men sometimes attribute infertility to a lack of compatibility between partners [35]. Divorce and re-marriage are relatively common [27]; thus, women may have “resolved” infertility through re-partnering. The present study does not account for possible changes in partnerships during the study period, as these data were not available.

Infertility measures may also be vulnerable to recall bias. Recalling past experiences using a two-year time marker may not be salient in this population. Another study using UTHA cohort data suggested that recalling the timing of specific events may be inaccurate in this low-numeracy population [37]. It is possible that women reported trying for two years to become pregnant, but, in fact, they had tried for a shorter amount of time. Qualitative evidence from this study population indicates that women may consider themselves infertile, and be perceived by others as being infertile, after a much shorter period of time due to social pressures and norms around childbearing [35].

The measure of infertility we used, and, indeed, nearly all self-reported infertility measures, also implies that women will readily differentiate between periods of time when they ‘tried’ to become pregnant and when they did not ‘try.’ We know that pregnancy intentions can change over relatively short periods of time [14] and that ambivalence around pregnancy is common in Malawi [21]; thus, women may have responded based on an overall, generalized assessment of a two-year period in their life, even if they were not trying to get pregnant (or exposed to pregnancy) for the entire duration of two years. Although the infertility question specifies exposure to pregnancy by asking if the woman “tried” to become pregnant, the concept of trying for pregnancy is highly subjective and relies on women accurately recalling and reporting many different components of sexual behavior.

Additionally, contraceptive use is excluded from this analysis because there was no measure of continuous contraceptive use in the data. However, given the commonality of contraceptive use in the cohort overall [38], it is likely that women’s exposure to the possibility of becoming pregnant was less than the time presumed in the analysis (i.e., the length of time a woman persisted in the cohort). Although many methods of contraception are vulnerable to imperfect use or intentionally inconsistent use (e.g., injectables) [39], some women likely used these methods efficaciously or used methods of contraception with close to perfect efficacy (e.g., the implant) [40]. These women may have been almost entirely invulnerable to pregnancy for part of or even for the entirety of the follow up period; however, we are unable to assess this from the available data. Thus, contracepting women are still included in the denominator. To assess the potential effects of sterilization, women who were sterilized after Wave 1 were examined, finding that more than half reported a new pregnancy before they were sterilized, justifying inclusion of women who were sterilized during the follow up period in the denominator.

Given the assumptions and likelihood that not everyone in the denominator was exposed to equal risk of becoming pregnant, it is notable that 65% of women still reported a pregnancy after Wave 1. With better measures of pregnancy exposure (e.g., longitudinal measures of contraceptive use and sexual frequency), it is possible that the pregnancy rate would have been even higher by removing from the denominator women who were not susceptible to pregnancy or adjusting for the length of exposure to the risk of pregnancy. There is also a strong possibility that pregnancies were under-reported in the data due to early miscarriage. Upwards of 20–30% of pregnancies result in a miscarriage [41, 42] and women may have a more difficult time recalling attributes of a pregnancy that did not result in a live birth [43]. It is also possible that some pregnancies were miscarried before the woman knew she was pregnant; these pregnancies would not be reported, contributing to a possible under-estimation of the number of women who became pregnant in the cohort.

Differential exposure to pregnancy due to contraceptive use between the women who had and had not experienced infertility could also potentially contribute to our finding of no difference between reporting a subsequent pregnancy in these groups. However, further analyses comparing desire for a/another child, which may be a proxy for contraceptive non-use, shows that similar proportions of women who had and had not experienced infertility wanted a/another child. This may suggest similar exposure to pregnancy throughout the study period between these groups.

An additional consideration is the definition of infertility that we used–two years without conceiving–which differs from the clinical and WHO standard definition of one year without conceiving [28]. This may have impacted our findings in two ways. First, it could mean that our results are conservative. Some respondents may have not conceived after one year, but conceived between one and two years (and thus, they would have met the clinical definition for infertility, but not be counted in our measure). A second implication of our measure is that it may be subject to greater recall bias than a one-year definition, as it is more difficult to recall a longer period of time [37].

In this study, we found that the LMUP was skewed toward intended pregnancies and women rarely reported the middle response options on the LMUP survey items (e.g., pregnancy happened at *not quite* the right time). The LMUP findings here differed from a previous application of the LMUP in Malawi, which observed more nuance in the measure [24]. Hall (2013) found that the median LMUP score was 6.0 (range = 0–12) among the 125 women surveyed, which is lower than the median score of 10 in the present study. However, Hall (2013) examined the LMUP among a smaller population of women in Malawi (N = 125) who were, on average, younger than the women in the present study (mean age of 24 vs. 27 years in the present study) and less likely to be married (80% vs. 93% in the present study). In the Malawian context, where childbearing is expected within marriage [27], it is likely that unmarried women have less intended pregnancies. Additionally, 71% of women in the present study reported prospectively that they wanted a/another child at Wave 1, which may contribute to the overall finding of high levels of pregnancy intendedness reported retrospectively.

### Conclusion

Despite limitations, this study fills an important gap in the existing literature by providing empirical evidence that women may experience both infertility and unintended pregnancy within their reproductive life course and that they may also experience an unintended pregnancy *after* experiencing infertility. This finding is critical for public health and reproductive justice, as it indicates not only the continued importance of addressing infertility and unintended pregnancy, but also the importance of examining the extent to which these diverse reproductive experiences may be experienced intermittently within an individual’s reproductive life course. The reproductive life course is complex–individuals may experience a range of events, including infertility, intended fertility, and unintended fertility. Integrating reproductive health services will better meet the needs of women by acknowledging and addressing these complexities. Due to data limitations and the quest to demonstrate specific causal relationships, most studies aim to isolate single reproductive outcomes; however, a more holistic examination better approximates women’s lived experiences as they navigate reproduction to inform public health programs and policies that will better meet reproductive health needs broadly.

Several conclusions can be drawn from this study with regard to the measurement and interpretation of infertility data and family planning programs. In terms of measurement, researchers should consider various definitions of infertility within epidemiology, medicine, and demography, as well as definitions that are meaningful for the study population. The latter is particularly important when infertility is highly stigmatized and people may consider themselves to be infertile even when they don’t meet the standard definitions (i.e., 1–2-year duration of exposure to pregnancy). Researchers must also consider the complex factors that inform an individual’s self-reported response to an infertility survey question, including how they might define actively and consistently ‘trying’ to become pregnant and using/not using contraceptive methods consistently. Evidence from this and other populations indicate that contraceptive behavior does not necessarily align with fertility preferences, nor indicate that contraceptive use is used according to clinical guidelines even when a pregnancy is not desired [39]. Lastly, there is a need for longitudinal data that allows the examination of the reproductive life course and how the meaning and consequences of reproductive experiences may differ based preceding or proceeding experiences [44].

This study found that infertility and unintended pregnancy are not mutually exclusive experiences. Similar rates of pregnancy between women regardless of whether or not they reported an experience of infertility suggests that all women, even those who have experienced infertility or difficulty conceiving, should be included in family planning programs and research that aims to enhance women’s reproductive autonomy. One way to ensure that women who have experienced infertility are included is to reexamine how we calculate important measures in family planning, such as unmet need for contraception. This measure is often used to evaluate the success of family planning programs, and it currently excludes those who meet the criteria for infertility or who self-identify as infertile [45, 46]. Further research is needed to better identify women who may have experienced infertility, but are still at risk of unintended pregnancy. Excluding women who have experienced infertility, either explicitly or inadvertently, from family planning programs may prevent women from achieving their reproductive goals.

## Supporting information

S1 Table Wave 1 characteristics of the pregnancy incidence analytic sample (N = 776) compared to those excluded due to lack of follow up (n = 94) or missing data (n = 55). (DOCX)

S2 Table Wave 1 characteristics of the pregnancy intentions sample (N = 375) compared to those excluded because they did not participate in Wave 5. (DOCX)

S3 Table LMUP items back-translated from Chichewa to English. (DOCX)

S4 Table Survivor time (not reporting a pregnancy) for each wave following Wave 11. (DOCX)

S5 Table Distribution of LMUP items by reported infertility at Wave 1 (N = 375). (DOCX)

S1 Fig Kaplan-Meier survival estimates comparing time-to-pregnancy (measured in waves) between women who did and did not report ever experiencing infertility at Wave 11. (DOCX)
