# Supplementary material for: Pregnancy and pregnancy intention after experiencing infertility: A longitudinal study of women in Malawi
Source: PLOS Glob Public Health. 2023 Nov 14;3(11):e0001646. doi: 10.1371/journal.pgph.0001646 (PMC10645290; doi:10.1371/journal.pgph.0001646)
Supplement: S1 Table — (DOCX) [file pgph.0001646.s001.docx]

**S1 Table.** Wave 1 characteristics of the pregnancy incidence analytic sample (N=776) compared to those excluded due to lack of follow up (n=94) or missing data (n=55)

|  | **Analytic sample**  **%/mean (range)** | **Excluded**  **%/mean (range)** | **Excluded sample N^1^** |
| --- | --- | --- | --- |
| **Reported a new pregnancy after W1** |  |  | 50 |
| Yes | 64.8% | 62.0% |  |
| No | 35.2% | 38.0% |  |
| **Infertility** |  |  | 113 |
| Yes | 19.7% | 22.1% |  |
| No | 80.3% | 77.9% |  |
| **Relationship status** |  |  | 141 |
| Married/cohabiting | 92.1% | 70.2% |  |
| Not married or cohabiting | 7.9% | 29.8% |  |
| **Age (mean (range))** | 26.6 (14-40) | 25.5 (14-41) | 149 |
| **Age group** |  |  | 149 |
| 14-19 | 12.2% | 26.2% |  |
| 20-24 | 31.6% | 25.5% |  |
| 25-29 | 21.4% | 14.8% |  |
| 30-34 | 20.0% | 19.5% |  |
| 35-41 | 14.8% | 14.1% |  |
| **STI history** |  |  | 145 |
| Yes | 10.4% | 7.6% |  |
| No | 89.6% | 92.4% |  |
| **Number of pregnancies (mean (range))** | 3.1 (0-12) | 2.7 (0-12) | 143 |
| **Number of pregnancies** |  |  | 143 |
| None | 7.2% | 23.8% |  |
| 1 | 16.6% | 14.0% |  |
| 2 | 20.0% | 12.6% |  |
| 3 | 17.8% | 13.3% |  |
| 4+ | 38.4% | 36.4% |  |
| **Number of living children (mean (range))** | 2.5 (0-8) | 2.1 (0-6) | 149 |
| **Number of living children** |  |  | 149 |
| None | 10.3% | 29.5% |  |
| 1 | 20.2% | 16.1% |  |
| 2 | 24.6% | 16.1% |  |
| 3 | 18.3% | 11.4% |  |
| 4+ | 26.6% | 26.9% |  |
| **Desire for another child (ever)** |  |  | 145 |
| Yes | 70.8% | 67.6% |  |
| No | 29.3% | 32.4% |  |
| **Years of education (mean (range))** | 5.2 (0-12) | 5.5 (0-12) | 145 |
| **Years in cohort (mean (range))** | 4.3 (1.7-5.1) | 4.3 (1.8-5.1) | 49 |

^1^Sample size varies for those excluded based on available data
