# Supplementary material for: Pregnancy and pregnancy intention after experiencing infertility: A longitudinal study of women in Malawi
Source: PLOS Glob Public Health. 2023 Nov 14;3(11):e0001646. doi: 10.1371/journal.pgph.0001646 (PMC10645290; doi:10.1371/journal.pgph.0001646)
Supplement: S2 Table — (DOCX) [file pgph.0001646.s002.docx]

**S2 Table.** Wave 1 characteristics of the pregnancy intentions sample (N=375) compared to those excluded because they did not participate in Wave 5

|  | **Analytic sample (N=375)** | **Excluded**  **(N=128)** |
| --- | --- | --- |
|  | **%/mean(range)** | **%/mean(range)** |
| **Infertility** |  |  |
| Yes | 18.4% | 23.4% |
| No | 81.6% | 76.6% |
| **Relationship status** |  |  |
| Married/cohabiting | 92.8% | 89.1% |
| Not married or cohabiting | 7.2% | 10.9% |
| **Age (mean (range))** | 26.0 (15-39) | 24.8 (14-39) |
| **Age group** |  |  |
| 14-19 | 12.3% | 21.8% |
| 20-24 | 37.3% | 35.2% |
| 25-29 | 19.7% | 16.4% |
| 30-34 | 17.1% | 18.0% |
| 35-41 | 13.6% | 8.6% |
| **STI history** |  |  |
| Yes | 7.5% | 13.3% |
| No | 92.5% | 86.7% |
| **Number of pregnancies (mean (range))** | 2.8 (0-10) | 2.2 (0-8) |
| **Number of pregnancies** |  |  |
| None | 8.0% | 18.8% |
| 1 | 20.5% | 21.9% |
| 2 | 22.1% | 21.1% |
| 3 | 16.3% | 17.2% |
| 4+ | 33.1% | 21.1% |
| **Number of living children (mean (range))** | 2.3 (0-8) | 1.9 (0-6) |
| **Number of living children** |  |  |
| None | 10.4% | 21.1% |
| 1 | 23.2% | 26.6% |
| 2 | 27.5% | 21.1% |
| 3 | 16.5% | 13.3% |
| 4+ | 22.4% | 18.0% |
| **Desire for another child (ever)** |  |  |
| Yes | 76.0% | 79.7% |
| No | 24.0% | 20.3% |
| **Years of education (mean (range))** | 4.9 (0-12) | 5.9 (0-12) |
