# Supplementary material for: Pregnancy and pregnancy intention after experiencing infertility: A longitudinal study of women in Malawi
Source: PLOS Glob Public Health. 2023 Nov 14;3(11):e0001646. doi: 10.1371/journal.pgph.0001646 (PMC10645290; doi:10.1371/journal.pgph.0001646)
Supplement: S3 Table — (DOCX) [file pgph.0001646.s003.docx]

**S3 Table.** LMUP items back-translated from Chichewa to English

| 1. **In the month I became pregnant, I** |
| --- |
| Always used contraception (0) |
| Always used contraception, but knew that the method had failed (1) |
| Used contraception, but not every time (1) |
| Was not using contraception (2) |
| 1. **Pregnancy happened at** |
| Wrong time (0) |
| Not quite right time (1) |
| The right time (2) |
| 1. **Just before I became pregnant, I** |
| Did not intend to get pregnant (0) |
| Intention kept changing (1) |
| Intended to get pregnant (2) |
| 1. **Just before I became pregnant, I** |
| Did not want a baby (0) |
| Mixed feelings about a baby (1) |
| Wanted to have a baby (2) |
| 1. **Before I became pregnant…** |
| My partner and I never discussed having children together (0) |
| My partner and I discussed children, but did not agree I should become pregnant (1) |
| My partner and I agreed for me to become pregnant (2) |
| 1. **Before becoming pregnant did you do anything to improve your health (e.g., took iron, saved money, ate healthily, sought advice from healthcare worker, or something else?)** |
| No (0) |
| Yes (2) |
