# Supplementary material for: Pregnancy and pregnancy intention after experiencing infertility: A longitudinal study of women in Malawi
Source: PLOS Glob Public Health. 2023 Nov 14;3(11):e0001646. doi: 10.1371/journal.pgph.0001646 (PMC10645290; doi:10.1371/journal.pgph.0001646)
Supplement: S4 Table — (DOCX) [file pgph.0001646.s004.docx]

S4 Table. Survivor time (not reporting a pregnancy) for each wave following Wave 1^1^

|  | **Beginning total** | **Fail** | **Net lost** | **Survivor function** | **95% CI** |
| --- | --- | --- | --- | --- | --- |
| **Total** |  |  |  |  |  |
| **Wave 3** | 477 | 211 | 21 | 0.56 | 0.51 - 0.60 |
| **Wave 4** | 245 | 56 | 17 | 0.43 | 0.38 - 0.47 |
| **Wave 5** | 172 | 46 | 126 | 0.32 | 0.27 - 0.36 |
| **No infertility** |  |  |  |  |  |
| **Wave 3** | 386 | 175 | 15 | 0.55 | 0.50 - 0.59 |
| **Wave 4** | 196 | 46 | 13 | 0.42 | 0.37 - 0.47 |
| **Wave 5** | 137 | 39 | 98 | 0.30 | 0.25 - 0.35 |
| **Infertility** |  |  |  |  |  |
| **Wave 3** | 91 | 36 | 6 | 0.60 | 0.50 - 0.70 |
| **Wave 4** | 49 | 10 | 4 | 0.48 | 0.37 - 0.58 |
| **Wave 5** | 35 | 7 | 28 | 0.38 | 0.28 - 0.49 |

^1^Failure indicates reporting a new pregnancy, upon which the participant exits the risk set. Net lost indicates women who were censored due to sterilization.
