# Supplementary material for: Pregnancy and pregnancy intention after experiencing infertility: A longitudinal study of women in Malawi
Source: PLOS Glob Public Health. 2023 Nov 14;3(11):e0001646. doi: 10.1371/journal.pgph.0001646 (PMC10645290; doi:10.1371/journal.pgph.0001646)
Supplement: S5 Table — (DOCX) [file pgph.0001646.s005.docx]

**S5 Table.** Distribution of LMUP items by reported infertility at Wave 1 (N=375)

|  | **Total sample (N=375)** | **No infertility W1 (N=306)** | **Infertility W1 (N=69)** |
| --- | --- | --- | --- |
| 1. **In the month I became pregnant, I** |  |  |  |
| Always used contraception (0) | 5.3% | 5.6% | 4.4% |
| Used contraception, but not every time (1) | 3.7% | 3.6% | 4.4% |
| Was not using contraception (2) | 90.9% | 90.9% | 91.3% |
| 1. **Pregnancy happened at** |  |  |  |
| Wrong time (0) | 15.2% | 14.4% | 18.8% |
| Not quite right time (1) | 3.5% | 3.9% | 1.5% |
| The right time (2) | 81.3% | 81.6% | 79.7% |
| 1. **Just before I became pregnant, I** |  |  |  |
| Did not intend to get pregnant (0) | 17.1% | 16.3% | 20.3% |
| Intention kept changing (1) | 2.1% | 2.0% | 2.9% |
| Intended to get pregnant (2) | 80.8% | 81.7% | 76.8% |
| 1. **Just before I became pregnant, I** |  |  |  |
| Did not want a baby (0) | 18.4% | 18.0% | 20.3% |
| Mixed feelings about a baby (1) | 0.3% | 0.3% | 0.0% |
| Wanted to have a baby (2) | 81.3% | 81.7% | 79.7% |
| 1. **Before I became pregnant…** |  |  |  |
| My partner and I never discussed children (0) | 15.8% | 15.4% | 17.4% |
| My partner and I discussed children (1) | 2.4% | 2.3% | 2.9% |
| My partner and I agreed to have a child (2) | 81.8% | 82.3% | 79.7% |
| 1. **Before becoming pregnant did you do anything to improve your health (e.g., took iron, saved money, ate healthily, sought advice from HC worker)** |  |  |  |
| No (0) | 65.1% | 63.4% | 72.5% |
| Yes (2) | 34.9% | 36.6% | 27.5% |
