# Supplementary material for: Pregnancy and pregnancy intention after experiencing infertility: A longitudinal study of women in Malawi
Source: PLOS Glob Public Health. 2023 Nov 14;3(11):e0001646. doi: 10.1371/journal.pgph.0001646 (PMC10645290; doi:10.1371/journal.pgph.0001646)
Supplement: S1 Fig — (DOCX) [file pgph.0001646.s006.docx]

S1 Fig. Kaplan-Meier survival estimates comparing time-to-pregnancy (measured in waves) between women who did and did not report ever experiencing infertility at Wave 1^1^

^1^On the x-axis: 0=Wave 1; 1=Wave 3; 2=Wave 4; 3=Wave 5

^2^The y-axis is the survival function
